# Mechanism of elastic energy storage of honey bee abdominal muscles under stress relaxation

**DOI:** 10.1093/jisesa/iead026

**Published:** 2023-05-08

**Authors:** Zhizhong Deng, Yuling Zhang, Shaoze Yan

**Affiliations:** Division of Intelligent and Biomechanical Systems, State Key Laboratory of Tribology in Advanced Equipment, Department of Mechanical Engineering, Tsinghua University, Beijing 100084, PR China; Division of Intelligent and Biomechanical Systems, State Key Laboratory of Tribology in Advanced Equipment, Department of Mechanical Engineering, Tsinghua University, Beijing 100084, PR China; Division of Intelligent and Biomechanical Systems, State Key Laboratory of Tribology in Advanced Equipment, Department of Mechanical Engineering, Tsinghua University, Beijing 100084, PR China

**Keywords:** honey bee abdominal muscle, stress relaxation, mechanism model, cross-bridge, elastic energy

## Abstract

Energy storage of passive muscles plays an important part in frequent activities of honey bee abdomens due to the muscle distribution and open circulatory system. However, the elastic energy and mechanical properties of structure in passive muscles remain unclear. In this article, stress relaxation tests on passive muscles from the terga of the honey bee abdomens were performed under different concentrations of blebbistatin and motion parameters. In stress relaxation, the load drop with the rapid and slow stages depending on stretching velocity and stretching length reflects the features of myosin–titin series structure and cross-bridge–actin cyclic connections in muscles. Then a model with 2 parallel modules based on the 2 feature structures in muscles was thus developed. The model described the stress relaxation and stretching of passive muscles from honey bee abdomen well for a good fitting in stress relaxation and verification in loading process. In addition, the stiffness change of cross-bridge under different concentrations of blebbistatin is obtained from the model. We derived the elastic deformation of cross-bridge and the partial derivatives of energy expressions on motion parameters from this model, which accorded the experimental results. This model reveals the mechanism of passive muscles from honey bee abdomens suggesting that the temporary energy storage of cross-bridge in terga muscles under abdomen bending provides potential energy for springback during the periodic abdomen bending of honey bee or other arthropod insects. The finding also provides an experimental and theoretical basis for the novel microstructure and material design of bionic muscle.

## Introduction

Frequent movements of the honey bee abdomen, such as pumping in feeding (Zhao et al. 2019), bending in flying (Taylor et al. 2013, Zhao et al. 2015), and swinging (Klein et al. 2018) in communication, require sufficient energy supply to the muscles among segments (Zhang et al. 2019, 2021, 2022). However, the adjoining-exoskeleton distribution and open circulatory system of abdominal muscles provide a slow material exchange condition (Carreck et al. 2013), causing a lack of dynamophore to support the whole process of abdominal movement. Thus, there should be structures for energy storage during the cyclical movement of the honey bee abdomen. It has been found that muscles from frogs (Linari et al. 2003) and mice (Lemaire et al. 2019) stored energy during the active stretch. However, there is neither a definite structure for energy storage proved nor a corresponding energy storage model established on passive muscles for explaining the probable energy storage structure on the terga muscles of honey bee abdomens.

Sufficient researches on muscle structure have distinguished that myosin, actin (Hanson and Huxley 1953, Huxley HE 1957) and titin (Maruyama 1976, Wang et al. 1979) are the 2 most masses of structural proteins. Besides, there are complex biomechanical interactions among the 2 proteins (Staple et al. 2009, Delmotte et al. 2011, Banigan et al. 2013, Dasanayake and Carlsson 2013). Specifically, myosin combines with titin (Maruyama et al. 1981, Herzog 2014) in series to form the frame structure of muscles, while actin also connects to titin. What’s more, actin produces interaction (Huxley AF 1957, Huxley and Brown 1967, Haselgrove and Huxley 1973) with parallel myosin through periodic motion (Jarvis et al. 2021) of cross-bridges. Although the difference between vertebrate and invertebrate muscles exists in the kinds and contents of proteins (Hooper et al. 2008), the myosin–titin series structure and cross-bridge–actin connection cycle are applied in both muscles (Nikonova et al. 2020). These structures constitute a biostructure base for energy storage.

There is an intricate conversion (Yamada 2017) among chemical, thermal, and mechanical energy in muscles. Muscles from frogs, mice, and rabbits have been observed storing energy during active movement by measuring heat, mechanical work, and given component concentration (Klip et al. 1981, Linari et al. 2003, Joumaa and Herzog 2013, Fukutani et al. 2017, Lemaire et al. 2019). Moreover, active muscles from moths (George et al. 2013) have been found a correlation between energy storage and mass transfer between filaments by force measurement and X-ray diffraction. The mechanism of contractility activated by calcium ions in honey bee abdominal muscle has been revealed, and the mechanical property of contracting muscle has also been measured (Zhang et al. 2021). However, these researches focusing on active muscles are unable to represent muscles from honey bee abdominal terga because they are always passively stretched (Taylor et al. 2013, Zhao et al. 2015). Besides, there is no experimental design to prove the energy storage capacity for a certain structure in muscles by applying targeted chemicals.

In laser trap experiments, asynchronous motion (Plaçais et al. 2009) of different cross-bridge has been found by vibration frequency measurement for cross-bridge groups. Then nonlinear elasticity (Kaya and Higuchi 2010) of a single myosin molecule has been detected by measuring the stiffness of cross-bridge in various states, as the same conclusion in other experiments (Persson et al. 2013, Linari et al. 2020). Therefore, the cross-bridge is potential to store elastic energy except the frame structure consisting of myosin and titin, which can change stiffness (DuVall et al. 2017; Nishikawa et al. 2020a, 2020b) in different states. To obtain the mechanical properties of passive muscles, it is appropriate to conduct stress relaxation tests on muscles from honey bee abdominal terga. Because the mechanical properties of various structural proteins would be reflected in the relaxation curve characteristics for the sudden deformation in stress relaxation tests (Linke and Leake 2004, Zhao et al. 2010, Bruno et al. 2011, Caporizzo et al. 2020). Besides, available analysis and modeling (Meyer et al. 2011, Wheatley et al. 2016, De Vita et al. 2017, Chiu et al. 2020, Wheatley 2020, Zhang et al. 2023) of passive muscles regard it as an entirety, which might not demonstrate different mechanical properties of structural proteins.

In the present study, we applied stress relaxation tests with different motion parameters on muscles from the honey bee abdominal terga by applying blebbistatin (Lemaire et al. 2019) to particularly target to bind cross-bridge. Based on the stress relaxation results, we improved the model, which was divided into different modules according to the characteristics of myosin–titin series structure and cross-bridge-actin connection in muscle structure. By discussing these results and the model, the mechanism of passive muscles during stress relaxation and the capability of elasticity energy storage of cross-bridge in passive muscles from honey bee abdominal terga was deduced.

## Materials and Methods

### Experimental Animals

The species involved in this study were adult foraging worker bees (*Apis mellifera* Linnaeus), raised in the intelligent biomechanical laboratory of Tsinghua University in Beijing, China (40.00°N, 116.33°E). Honey bees were fed with honey and water regularly and kept in a cage consisting of a 120 × 40 × 60 cm transparent glass cover and a 50 × 40 × 26 cm hive at room temperature. This experiment neither involved endangered or protected species nor required special permission.

### Sample Preparation

**Fig. 1.**
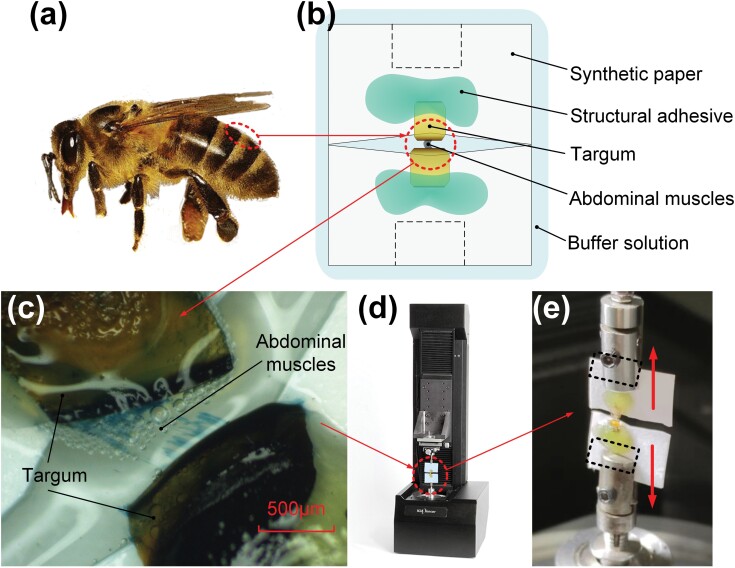
Sample of abdominal muscles from the honey bee. (a) The selected area of terga from honey bee to make sample. (b) The composition of sample and distribution of the composition. (c) The observation of abdominal muscles on the sample under a stereoscope. (d) Micro-nano Tensile System. (e) The sample in the experiment and the clamping area of sample for collets (black dashed bordered rectangle in (b) and (e)).

Honey bee abdomens were dissected in buffer solution A (NaCl 130 mmol/l, KCl 6 mmol/l, CaCl_2_ 0.11 mmol/l, MgCl_2_ 4 mmol/l, glucose 25 mmol/l, Na_2_HPO_4_ 10 mmol/l, sucrose 125 mmol/l, pH 6.7) at 4 °C to remove viscera and get the selected terga area of the abdomen in Fig. 1a. Then the intersegmental membrane between 2 exoskeletons was cut off to remain muscles solidly connected to exoskeletons under a microscope (Axiostar Plus, Zeiss, Germany) in buffer solution A at room temperature. Polypropylene synthetic paper (PPSP) (Glossy PPSP, Liwu, China), as the base of the sample, was prepared in a 1 × 1.5 cm rectangle with a 1.5-mm crevice in the middle. After surrounding the PPSP with buffer solution A, muscles with 2 exoskeletons connected were transferred to the crevice. Then, 2 exoskeletons were moved onto segments on the sides of the crevice with the integrity of muscles. In the final operation, Acrylic Structural Adhesive (DP810 Tan, 3M, USA) was applied to the gap between PPSP and exoskeletons on the side away from muscles. Samples were placed in buffer solution A for 20 min to set the adhesive and then complete sample production as Fig. 1b. Samples were placed in buffer solution X (NaCl 130 mmol/l, KCl 6 mmol/l, CaCl_2_ 0.11 mmol/l, MgCl_2_ 4 mmol/l, glucose 25 mmol/l, Na_2_HPO_4_ 10 mmol/l, sucrose 125 mmol/l, 0.1% Janus green, pH 6.7) for 10 min to dye muscles and observed under a stereoscope to ensure the presence of fresh muscles on the sample in Fig. 1c.

### Experimental Equipment

Muscle deformation control and load measurement were realized by Micro-nano Tensile System (T150 UTM, Agilent, Santa Clara, USA) in Fig. 1d. Its maximum load is 500 mN with a load resolution of 50 nN, and the maximum displacement is ±1 mm with a displacement resolution of ~0.1 nm. The system consists of a control computer and an experimental device composed of 2 platforms. The upper platform contains a collet and motion drive, and the lower platform contains a collet and force sensor. The 2 segments of the sample were installed in 2 collets separately in Fig. 1e in stress relaxation tests.

### Stress Relaxation Tests

Before installing the sample, control group and experimental group were soaked separately in buffer solution B (NaCl 130 mmol/l, KCl 6 mmol/l, CaCl_2_ 5 mmol/l, MgCl_2_ 4 mmol/l, glucose 25 mmol/l, Na_2_HPO_4_ 10 mmol/l, sucrose 120 mmol/l, pH 6.7) and buffer solution C (NaCl 130 mmol/l, KCl 6 mmol/l, CaCl_2_ 5 mmol/l, MgCl_2_ 4 mmol/l, glucose 25 mmol/l, Na_2_HPO_4_ 10 mmol/l, sucrose 120 mmol/l, blebbistatin 10 μmol/l, pH 6.7) for 30 min at 4 °C to make the solution fully permeate the muscle. Adding blebbistatin to the sample in the experimental group was to make cross-bridge combine with blebbistatin specifically and to change the mechanical properties of cross-bridge. After installing the sample and snipping the lateral connection aside crevice, muscles should be prestretched. In prestretching, muscles were stretched with a stretching velocity of 0.01 mm/s and ended stretching with continuous load growth for 10 s to ensure the separation of the 2 honey bee abdominal terga. Prestretching aimed to make a certain tension on muscles at the beginning of tests. Two or 3 stress relaxation tests were performed on each sample with various stretching velocities of 0.01–0.25 mm/s and stretching lengths of 0.01–0.4 mm. The range of stretching velocity and length was below the limit of muscle bearing and above the minimum of no obvious result. Besides, muscles were kept fresh in the experiment by spraying corresponding buffer solution on muscles between 2 tests. We conducted 21 tests of 9 samples in the control group and 13 tests of 5 samples in the experimental group. The 3 characters of test number represented group (control or experimental), muscle number in the group, and test number of the muscle, respectively. For example, c11 and c12 were the first and second tests of the first sample in the control group. Motion parameters of each test were shown in Supplementary Table A1.

It is necessary to consider the influence of structural adhesive and synthetic paper on the experiment. The shear strength of structural adhesive used in this study was about 25 MPa at 24 °C, while the main component of synthetic paper was polypropylene, of which yield strength was about 30 MPa. The maximum load of muscle was less than 10mN, with the minimum bonding area to the adhesive of 3 × 1 mm and cross-sectional area in the synthetic paper of 10 × 0.1 mm. Hence, the maximum shear stress of adhesive and tensile stress of paper was 8.3 and 10 kPa, which was far less than 0.33% of 25 and 30 MPa. However, the ultimate strains of the adhesive and the paper were less than 50%, while the strain of muscles in the test was more than 10%. Therefore, the deformation of adhesive and synthetic paper was less than 1% of the deformation of muscles in the test with the same millimeter-scale size, which could be ignored.

### Two-Parallel-Module Model of Passive Muscle

We improved the model with 2 parallel modules to fit the passive behavior in the abdominal muscles of honey bees. One of the modules was passive viscoelastic module (PVM) composed of myosin and titin in series to represent the main structure of muscles. Another module was active elastic module (AEM) referring to the cyclical movement and nonlinear elasticity of cross-bridge.

In PVM, we used quasi-linear viscoelastic SYS (Van Loocke et al. 2008) to describe the structure composed of viscoelastic myosin and titin in series for the statistic of slow stress relaxation stage from tests. According to the SYS, the main stress of $\sigma_{j}(t)$ in direction *j* can be expressed as $\;\sigma_{j}(t)=\sigma_{j}^{e}(0+)G_{j}(t)+\underset{0}{\overset{t}{\int }}\;G_{j}(t-\tau )\frac{\partial \sigma_{j}^{e}[{\tilde{\varepsilon }}_{j}(\tau )]}{\partial \tau }d\tau$, where $\sigma_{j}^{e}=E_{1}\lambda_{j}$ is the elastic stress (*E*_1_ is the elastic coefficient), $G_{j}(t)$ is the relaxation function, and ${\tilde{\varepsilon }}_{j}=\ln(\lambda_{j})$ is the logarithmic strain (λ_*j*_ is the stain in direction *j*). Using a Prony series expansion, $G_{j}(t)=p_{\infty j}+\underset{i=1}{\overset{N}{\sum }}\;p_{ij}e^{-t/\tau_{ij}}$ and applying stretching length of loading process $\lambda_{j}(t)=\left\{ \begin{array}{l} \frac{\lambda_{0j}}{t_{0}}t\;(0<t<t_{0})\\ \lambda_{0j}\;(t_{0}\leq t)\; \end{array}\right.$ in $\sigma_{j}(t)$ leads to:


$$\begin{array}{l} \;\;\;\sigma_{j}(t)=p_{\infty j}E_{1}\lambda_{0j}+\underset{i=1}{\overset{N}{\sum }}\;p_{ij}E_{1}\frac{\lambda_{0j}}{t_{0}}\tau_{ij}(e^{\frac{t_{0}}{\tau_{ij}}}-1)e^{-\frac{t}{\tau_{ij}}},\;\;\;\;\;(t_{0}\leq t) \end{array}$$ (1)

where $\tau_{ij}$ and $p_{ij}$ are parameters related to muscle viscoelasticity. We assumed that the cross-sectional area of muscles was *S* and the main load of PVM on muscles was $F_{PVM}(t)=\sigma_{j}(t)S$. To ignore the higher-order infinitesimal, a 2-term Prony series was applied in $F_{PVM}(t)$ by taking *N* = 2. Besides, we considered that the load in measurement was along the main stress direction. Finally, the load fitting equation of PVM on relaxation stress can be expressed as:


$$\begin{array}{l} F_{PVM}(t)=C_{0}+C_{1}e^{-\frac{t}{\tau_{1}}}+C_{2}e^{-\frac{t}{\tau_{2}}},\;\;\;\;\;\;\;\;\;\;(t_{0}\leq t) \end{array}$$ (2)

where $C_{0}=p_{\infty j}E_{1}\lambda_{0j}S+C$ (*C* is a constant term coefficient) and $C_{i}=p_{ij}E_{1}\frac{\lambda_{0j}}{t_{0}}\tau_{ij}(e^{\frac{t_{0}}{\tau_{ij}}}-1)S$ (*i* = 1,2) are fitting parameters.

In AEM, the main characteristics were the cyclical movement and nonlinear elasticity of cross-bridge. According to the linear elasticity of cross-bridge in stretching (Kaya and Higuchi 2010, Linari et al. 2020), the elastic force of a single connected cross-bridge could be expressed as $f_{i}(\Delta l_{i})=\Delta l_{i}E_{2}$ (1 ≤ *i* ≤ *n*), where Δ*l*_*i*_ is the elastic deformation of cross-bridge in the direction of filament axis, *E*_2_ is the stiffness of cross-bridge, and *n* is the amount of connected cross-bridge. Considering the constant stretching velocity (*v*) in loading process and the time for stretching on one of the connected cross-bridges (Δ*t*_*i*_), the elastic deformation is expressed as $\Delta l_{i}=\Delta t_{i}v$. Furthermore, Δ*t*_*i*_ (1 ≤ *i* ≤ *n*) was uniformly distributed on interval range of [0, Δ*t*] for the asynchronous motion of different cross-bridge (Plaçais et al. 2009), where Δ*t* is the average time of contacting with actin in the cyclical movement of cross-bridge.

In stress relaxation of *t*_0_ < *t*, the cross-bridge in the elastic deformation of Δ*l*_*i*_ could keep deformation for Δ*t* − Δ*t*_*i*_ due to the cyclical movement leading to the elastic force:


$$\begin{array}{l} f_{i}(t)=f_{i}(t_{0})(1-\varepsilon ((t-t_{0})-(\Delta t-\Delta t_{i}))),\;(t_{0}\leq t<t_{0}+\Delta t) \end{array}$$ (3)

where ε(t) is Step Function. Thus, the sum force in AEM composed of elastic force in cross-bridges could be expressed as $F_{AEM}(t)=\underset{i=1}{\overset{n}{\sum }}\;f_{i}(t)$. Substituting Equation (3) and uniform distribution of Δ*t*_*i*_ in the sum force leads to:


$$\begin{array}{l} F_{AEM}(t)=A_{2}{\left(t-t_{0}\right)}^{2}+A_{1}(t-t_{0})+A_{0}\;(t_{0}\leq t<t_{0}+\Delta t) \end{array}$$ (4)

where $A_{2}=\frac{nvE_{2}}{2\Delta t}$, $A_{1}=-nvE_{2}$, and $A_{0}=\frac{\Delta tnvE_{2}}{2}$ are fitting parameters. According to the expression of *A*_2_, *A*_1,_ and *A*_0_, these fitting parameters basically depend on structure parameters *n*, *E*_2_, and Δ*t*. Thus, the influence of blebbistatin on cross-bridge in passive muscle can be expressed with the calculation of *n*, *E*_2_, and Δ*t*.

### Statistics and Data Processing

**Fig. 2.**
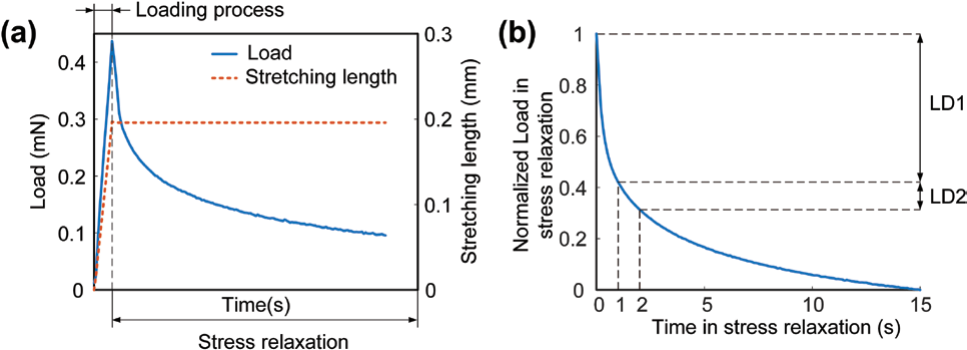
Stress relaxation curve from honey bee abdominal muscles. (a) Load-time and stretching length-time curves of stress relaxation test. (b) The normalized stress relaxation curve and load drops (LD) in 1 s of 2 characteristic processes (0–1 and 1–15 s in stress relaxation).

The stretching length and load of honey bee abdominal muscles at each time point under different motion conditions were recorded. Then the scatter diagrams of muscle stretching length-time and load-time were drawn. For example, the data of test c61 were drawn in Fig. 2a, and the process of the test was divided into loading process and stress relaxation according to the stretching length. The curve fitting in modeling analysis adopted exponential fitting using the Levenberg–Marquardt optimization algorithm for iterative fitting conducted in ORIGIN (OriginLab Inc., USA). The tolerance value of Chi SQR 10^−12^ was used to judge the convergence of fitting and coefficient *R*^2^ was used to judge the goodness of fit. Data and fitting parameters were analyzed using Excel 2019. Besides, all obtained *P* values are the result of 2-sided tests and the α was set at *P* = 0.05. Fitting parameters were expressed as means ± SE.

## Result

### Two Characteristic Stages of Stress Relaxation on Honey Bee Abdominal Muscles

**Fig. 3.**
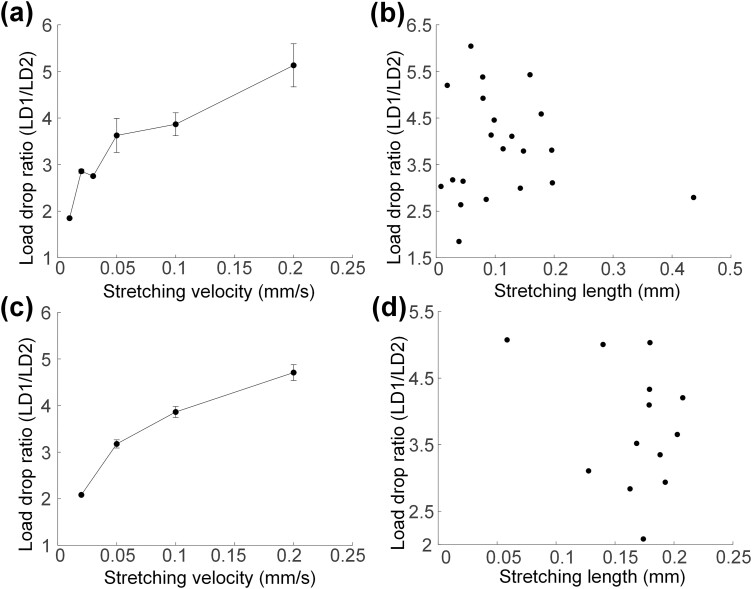
Correlation between the ratio of LD1 to LD2 and motion parameters. (a) Results on stretching velocity in control group. (b) Results on stretching length in control group. (c) Results on stretching velocity in experimental group. (d) Results on stretching length in experimental group.

The normalized stress relaxation curve in Fig. 2b showed that there was a rapid load drop at the beginning of stress relaxation, and then a slow load drop occurred after 1 s. To contrast the 2 processes of stress relaxation quantitatively, the load drop within 2 s from the start of stress relaxation was split into 2 parts with equal durations. The result showed the load drop (LD1) with time from 0 to 1 s was more than 1.85 times the load drop (LD2) with time from 1 to 2 s in the control group, while the minimum multiple was 2.08 in the experimental group. According to different slopes of load drop, the 2 processes were, respectively, named as the rapid and slow stress relaxation stages. The intersection of the 2 stages at 1 s in stress relaxation was the transition point. Specifically, the comparison of the ratio of the 2 above load drops in the control and experimental groups under different motion parameters was shown in Fig. 3. The ratio raised from 1.85 ± 0.00 to 5.13 ± 0.46 with the increase of stretching velocity from 0.01 to 0.2 mm/s in the control group (Fig. 3a), while in the experimental group (Fig. 3c), the ratio raised from 2.08 ± 0.00 to 4.7 ± 0.17 with the increase of stretching velocity from 0.02 to 0.2 mm/s. The load ratio shows a positive relationship with stretching velocity, and there is no significant difference between different samples (*P* < 0.05). Besides, the slope decreases with the increase of stretching velocity. However, the stretching length showed a low correlation to the ratio in Fig. 3b and d (*P* > 0.05). The result suggests that the influence of stretching velocity on the load drop in the 2 stages is different while the stretching length shows the influence on the 2 stages in the same degree.

**Fig. 4.**
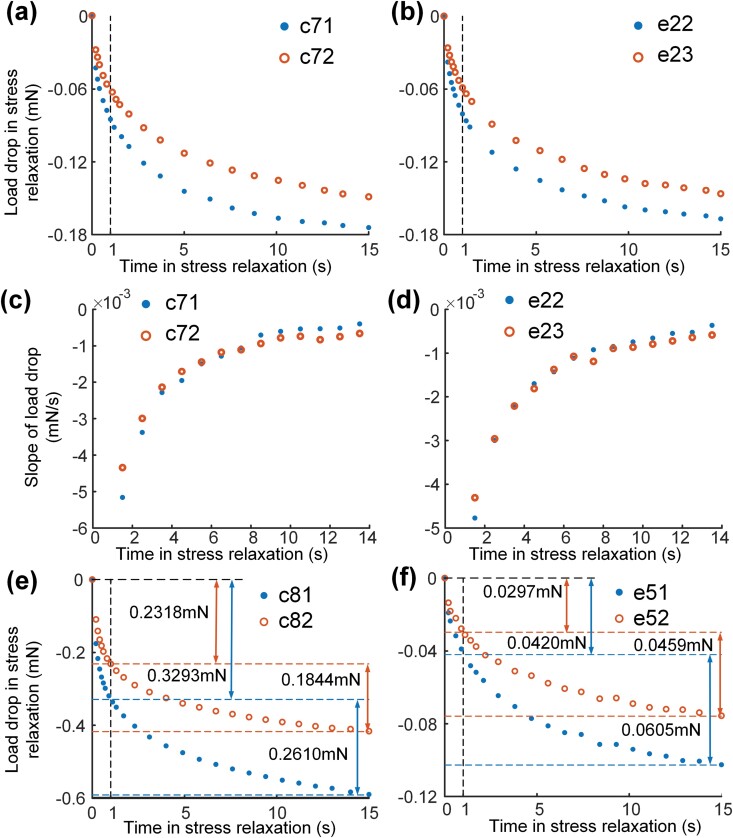
Contrast on load drops of 2 tests in the same sample. (a)–(d) Tests with the same stretching lengths and different velocity: Load drops in the whole process of stress relaxation in control (a) and experimental (b) groups; Slope contrast of load in slow stress relaxation stage (1–15 s in stress relaxation) in control (c) and experimental (d) groups. (e)–(f) Tests with the same stretching velocity and different lengths in the same sample; Load drops in control (e) and experimental (f) groups with the divide of load drops in rapid and slow stress relaxation.

To reveal the influence of stretching velocity on stress relaxation, we discussed the change trend of the load drop ratio in the 2 stress relaxation stages with different stretching velocities. The result showed that the ratio raised with the increase of stretching velocity in both groups, but the rate of raising became lower with high stretching velocity. It suggested that stretching velocity had an effect on the load drop in the rapid or slow stress relaxation stage and there was an upper limit on this effect. To identify the range of the effect, the stress relaxation curves of the same muscles with the same stretching length and different stretching velocities were performed in Fig. 4a and b. Results of the 2 groups showed that there was a larger load drop in the rapid stress relaxation stage with the higher stretching velocity. It illustrates that the stretching velocity has an effect on the rapid stress relaxation stage. Furthermore, the data of the slow stress relaxation stage in these curves were compared intuitively in Fig. 4c and d by calculating the slope of load drop. The load was recorded 10 times per second, so the average slope of load at *x*.5 s was solved through the 10 values from *x* s to (*x*+1) s, where *x* is the integer time in slow stress relaxation stage. It was distinct that slopes of load drop in the same muscles, but with different stretching velocities were basically in coincidence in the slow stress relaxation stage. The difference between the 2 slopes was less than 15% of the maximum when the time of stress relaxation came up to 1 s and the end of stress relaxation. Therefore, stretching velocity has little effect on the slow relaxation stage of muscles.

Stress relaxation curves of the same sample under the experimental condition of the same stretching velocity and different stretching lengths were shown in Fig. 4e and f. In the control group (Fig. 4e), with the same stretching velocity of 0.05 mm/s, the load released in the rapid stress relaxation stage with a stretching length of 0.178 mm was 1.42 times of another with a 0.113-mm stretching length. Similarly, the multiple of the load drop in the slow stress relaxation stage was 1.42, which was close to the stretching length ratio of 1.57. This proportional relationship between load released in the rapid/slow stress relaxation stage and stretching length was also satisfied in the experimental group (Fig. 4f). It suggests that stretching length has an effect on both the rapid and slow stress relaxation stages, and there is a certain linear relationship between the drop in the slow stress relaxation stage and the stretch length.

**Fig. 5.**
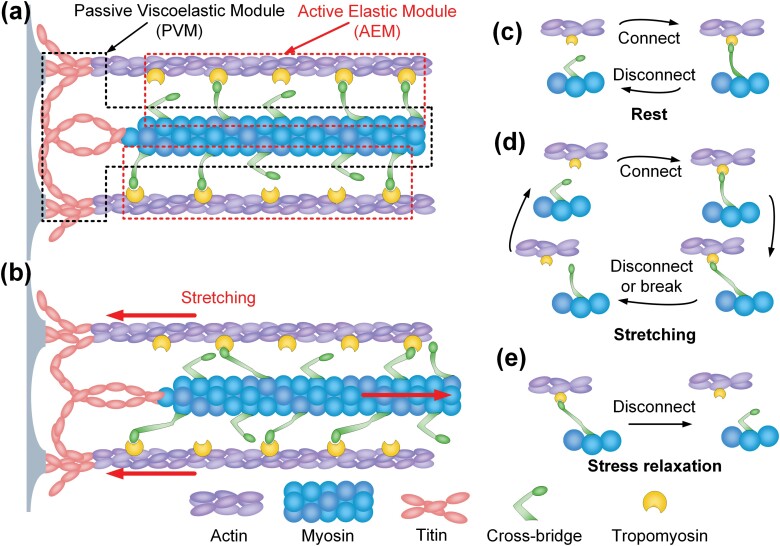
Deformation of structural protein of muscle in the control group test. (a) Sarcomere before loading process (in rest condition). (b) Sarcomere in loading process. (c) Cyclical combination between cross-bridge and actin in rest condition of sarcomere. (d) The detaching-combining-elongated-breaking loop of cross-bridge in loading process. (e) The breaking process of elongated cross-bridge in stress relaxation.

The stress relaxation curve is associated with the structure of muscles (Linke and Leake 2004, Abraham et al. 2013). The present results showed that there were 2 characteristic stages of stress relaxation in the honey bee abdominal muscles, which indicated that at least 2 kinds of characteristic structures existed in passive muscles during stretching and relaxing. Figure 5 showed the hypothesis on the deformation of structural protein of muscle in stress relaxation test. First, the load release in the rapid stress relaxation stage was in an immediate time (<1 s), which was similar to the characteristics of the cross-bridge in muscle structure. As mentioned above, the cycle time of cross-bridge is less than 1 s in active muscles (Kaya et al. 2017; Pertici et al. 2018), which could last more time in passive muscles (Fig. 5c). Furthermore, it is known that the cross-bridge has a linear elasticity in stretching through the optical tweezers experiment (Kaya and Higuchi 2010). Due to these properties of cross-bridge, when the thick moved relatively to thin filaments, as Fig. 5d showed that the cross-bridge could generate elastic deformation on the stage of binding to actin in the motion cycle. According to Fig. 5, the value of elastic deformation on each cross-bridge depended on the product of the relative velocity of filaments and duration of binding, while there was a positive correlation between the elasticity and deformation of cross-bridge. As soon as the end of binding in the motion cycle, Fig. 5e showed that the elastic potential energy stored in the cross-bridge would be released in stress relaxation. Thus, the rapid stress relaxation stage might include the release of the elastic potential energy of the cross-bridge. Second, stretching length had an active effect on both stages of stress relaxation, which could be an integral influence on muscles. The load relaxation of this part might be related to the series structure of myosin and titin in muscles in Fig. 5a and b. Therefore, muscles could be divided into 2 modules by various structural proteins. One is the viscoelastic myosin–titin series structure, and another is the autonomous cross-bridge–actin connection cycle. Our model was developed on the parallel structure of myosin–titin series structure and cross-bridge–actin connection cycle.

### Model Fitting

**Fig. 6.**
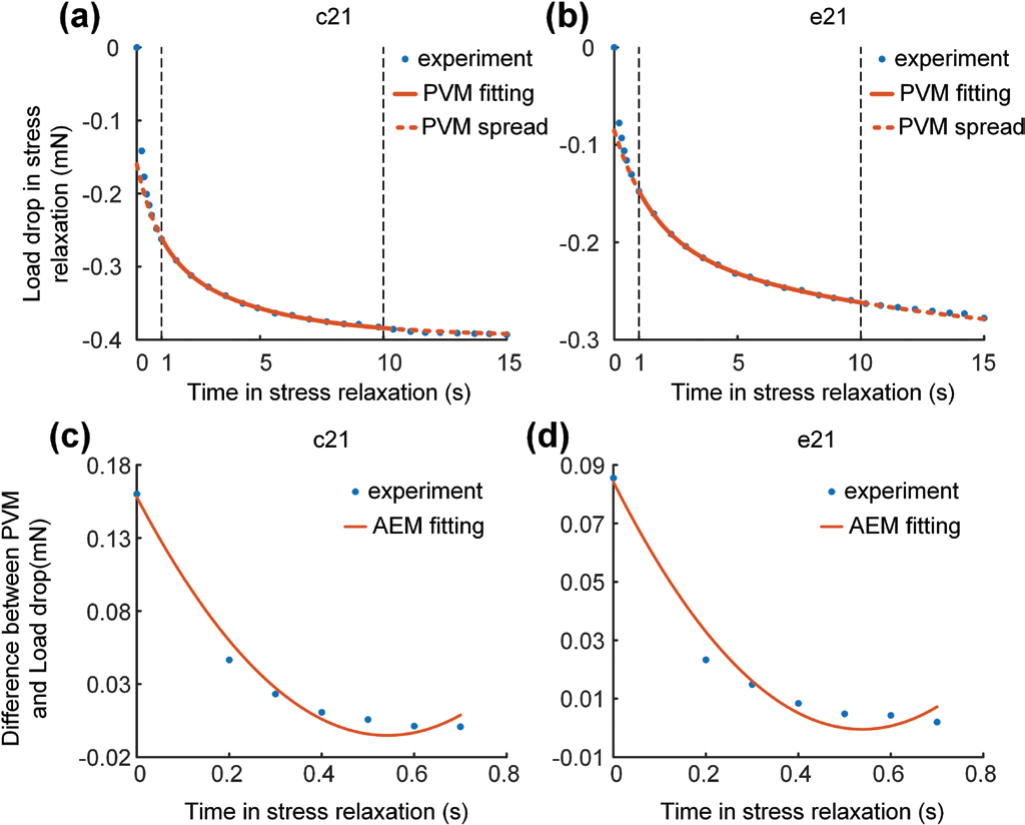
Fitting of PVM and AEM. (a), (b) Application of fitting PVM in 1~10 s of stress relaxation and expansion in the whole stress relaxation in control (a) and experimental (b) groups. (c), (d) Fitting of AEM on differences between experimental load drop data and the value calculated with fitting PVM (1–10 s in stress relaxation) in 0–1 s of stress relaxation in control (c) and experimental (d) groups.

As the model fitting, both PVM and AEM determined the load drop in the rapid stress relaxation stage, while only PVM determined the process of the slow stress relaxation stage. The data on slow stress relaxation in $t_{0}+1\;s\leq t\leq t_{0}+10\;s$ was chosen to be fit by PVM in Fig. 6a and b. The fitting was conducted in ORIGIN (OriginLab Inc., USA) by using the Levenberg–Marquardt optimization algorithm for iterative fitting. Analysis of fitting performed that the coefficient of determination (*R*^2^) was above 0.9978 at all stretching parameters and solution conditions.

The load fitting equation of PVM determined by slow stress relaxation in $t_{0}+1\;s\leq t\leq t_{0}+10\;s$ was applied to the whole process of stress relaxation in $t_{0}\leq t$ in Fig. 6a and b. As expected, experimental data were in good coincidence with the fitting equation in $t_{0}+0.5\;s\leq t$, but experimental data were above fitting equation in $t_{0}<t<t_{0}+0.5\;s$. Besides, the difference value between data and equation depended on stretching velocity. Theoretically, the difference value could be the load drop of AEM in stress relaxation.

Unexpectedly, Fig. 6a and b showed that experimental data were slightly less than fitting equation at the end of the experiment in several samples out of expectation. One reason was supposed to be water loss in the abdominal muscles of honey bees. Though solution was sprayed on muscles before each stretching, water was also lost over time in the experiment, especially in the condition of high stretching length and low stretching velocity. As water loss, muscle elasticity decreases (Ruiz-Ramírez et al. 2005, Lorenzo et al. 2019).

The fitting equation in AEM fitted to the difference value between experimental data and the fitting equation in PVM was shown in Fig. 6c and d. As expected, all difference value was of good fitting to the fitting equation in AEM with the coefficient of determination (*R*^2^) above 0.9950.

**Fig. 7.**
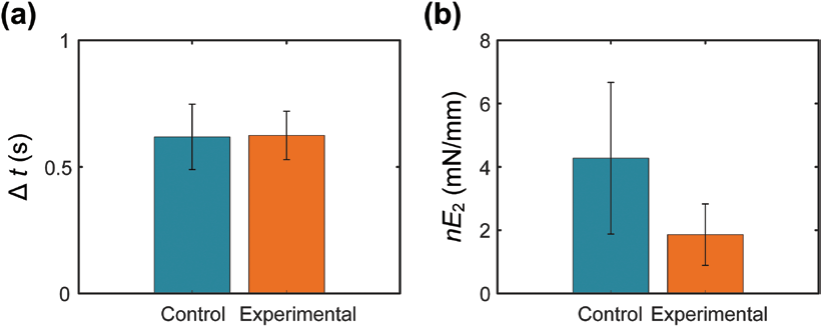
Comparison of fitting parameters in AEM between control and experimental group. (a) Average time of contacting with actin in the cyclical movement of cross-bridge (SE: Control = ±0.129s, Experimental = ±0.095s). (b) Product of stiffness and amount of cross-bridge (SE: Control= ±2.396 mN/mm, Experimental= ±0.971 mN/mm).

To identify the elastic deformation of cross-bridge, we compared fitting parameters in AEM of the experimental group with the parameters of the control group in Fig. 7. According to the expression of $A_{2}=\frac{nvE_{2}}{2\Delta t}$, $A_{1}=-nvE_{2}$, and $A_{0}=\frac{\Delta tnvE_{2}}{2}$, the approximate solution of average time of contacting with actin in the cyclical movement of cross-bridge could be expressed as $\Delta t=(-A_{1}/2A_{2}-2A_{0}/A_{1})/2$. Besides, the product of stiffness and amount of cross-bridge could be expressed as $nE_{2}=(2\Delta tA_{2}/v-A_{1}/v+2A_{0}/\Delta tv)/3$ with the known stretching velocity. Surprisingly, $nE_{2}$ showed a strong correlation with blebbistatin, while the average of Δt was the same value in both control (0.619±0.129s) and experimental (0.624±0.095s) groups. The value of $nE_{2}$ in the control group (4.275±2.396mN/mm) was approximately 2.30 times than in the experimental group (1.861±0.971mN/mm), and no significant differences between different samples were seen in stretching velocity and length (*P* < 0.05).

## Discussion

### Verification of the Model

To verify the correctness of the model, the model parameters fitted with the data of a certain test should be applied to the data of another test for the fit error (Peña et al. 2007, Meyer et al. 2011). However, fitting parameters in PVM and AEM depended on many factors, such as motion parameters, biological activities, and surroundings. Although motion parameters of stretching velocity (*v*) and stretching length $(\lambda_{0j})$ were definite values from statistics, parameters of $p_{ij},\;\;E_{i},\;\;S,\;\;and\;\;n$ were uncontrollable. For example, parameters on mechanical properties of muscle $\left(E_{i}\right)$ changed for the decreased cell activity with the time gone by and water loss (Lorenzo et al. 2019). Structural parameters of muscle (*S*, *n*) were various in different muscles. Due to the diversity of fitting parameters in different tests, using the model to calculate the theoretical value out of stress relaxation could be a way to verify the model (Wheatley 2020). Therefore, we calculated the theoretical load in the loading process with the obtained model and contrasted it with experimental data to verify the correctness of the model.

To calculate the theoretical load in the loading process, the load was divided into 2 parallel parts for PVM and AEM of the model. The first part of the load depending on PVM in the loading process could be calculated through Boltzmann superposition principle for quasi-linear viscoelastic and ramp loading process (Knauss and Zhao 2007). The relationship between $F_{PVM}(t)$ and stretching length *l*(*t*) could be expressed as:


$$\begin{array}{l} F_{PVM}(t)=Y(t)*dl(t) \end{array}$$ (5)

where *Y*(*t*) is stress relaxation coefficients. To get *Y*(*t*), applying step deformation to muscles was a direct method. However, the loading process in this study was a ramp loading process $l(t)=\left\{ \begin{array}{l} \frac{l_{0}}{t_{0}}t\;(0<t<t_{0})\\ l_{0}\;(t_{0}\leq t)\; \end{array}\right.$, where *l*_0_ is the maximum stretching length. Besides, it has been found that when $10t_{0}\leq t$ in ramp tests, the value of load could be approximately expressed as $F_{PVM}(t)\approx Y(t)l_{0}$ (Lee and Knauss 2000). According to these methods on relaxation of ramp tests, substituting equation of *l*(*t*) and $F_{PVM}(t)$ to Equation (5) and taking the derivative with respect to _t_ leads *Y*(*t*):


$$\begin{array}{l} Y^{\prime }(t)=\left\{ \begin{array}{l} \frac{F_{PVM}(t)}{l_{0}},\;(Nt_{0}<t\leq (t+t_{0})t_{0})\\ Y^{\prime }(t+t_{0})-\frac{t_{0}}{l_{0}}\frac{dF_{PVM}(t+t_{0})}{dt},\;(0\leq t) \end{array}\right. \end{array}$$ (6)

where *N* ≥ 10 and $Y^{\prime }(t)$ is the theoretical approximate value of Y(t). Substituting $Y^{\prime }(t)$ and l(t) in the loading process (0 < *t *< *t*_0_) to Equation (5) leads the theoretical approximate value of $F_{PVM}(t)$ to:


$$\begin{array}{l} {F^{\prime }}_{PVM}\left(t\right)=\frac{l_{0}}{t_{0}}\underset{0}{\overset{t}{\int }}\;Y^{\prime }(t-\tau )d\tau ,\;(0<t<t_{0}) \end{array}$$ (7)

The second part of the theoretical load in the loading process depended on AEM. As mentioned in AEM, $F_{AEM}(t)$, was a compound force composed of force between cross-bridge and actin. According to the cyclical movement of cross-bridge (Jarvis et al. 2021) and uniform distribution of time on various cross-bridge binding to actin (Plaçais et al. 2009), the average elastic deformation of cross-bridge could be expressed as:


$$\begin{array}{l} F_{AEM}\left(t\right)=\left\{ \begin{array}{l} n\underset{0}{\overset{1}{\int }}\;vtxE_{2}dx,\;(0<t<\Delta t)\\ n\underset{0}{\overset{1}{\int }}\;v\Delta txE_{2}dx,\;(\Delta t<t<t_{0}) \end{array}\right. \end{array}$$ (8a)

which could be expressed with the parameters of AEM as:


$$\begin{array}{l} F_{AEM}\left(t\right)=\left\{ \begin{array}{l} A_{0}\frac{t}{\Delta t},\;(0<t<\Delta t)\\ A_{0},\;(\Delta t<t<t_{0}) \end{array}\right. \end{array}$$ (8b)

where $A_{0}=\frac{\Delta tnvE_{2}}{2}$ is the fitting parameters in AME. However, the Tension Trigger of Micro-nano Tensile System was 100 μN causing that no load recorded at the beginning of the loading process. Hence, the first piece of Equation (8b) could be ignored. Finally, the theoretical value of load in the loading process $(F^{\prime }(t))$ could be calculated by solving the sum of ${F_{PVM}}^{\prime }(t)$ and $F_{AEM}(t)$, which is expressed as $\;F^{\prime }(t)={F^{\prime }}_{PVM}(t)+F_{AEM}(t),\;(0<t<t_{0})$.

**Fig. 8.**
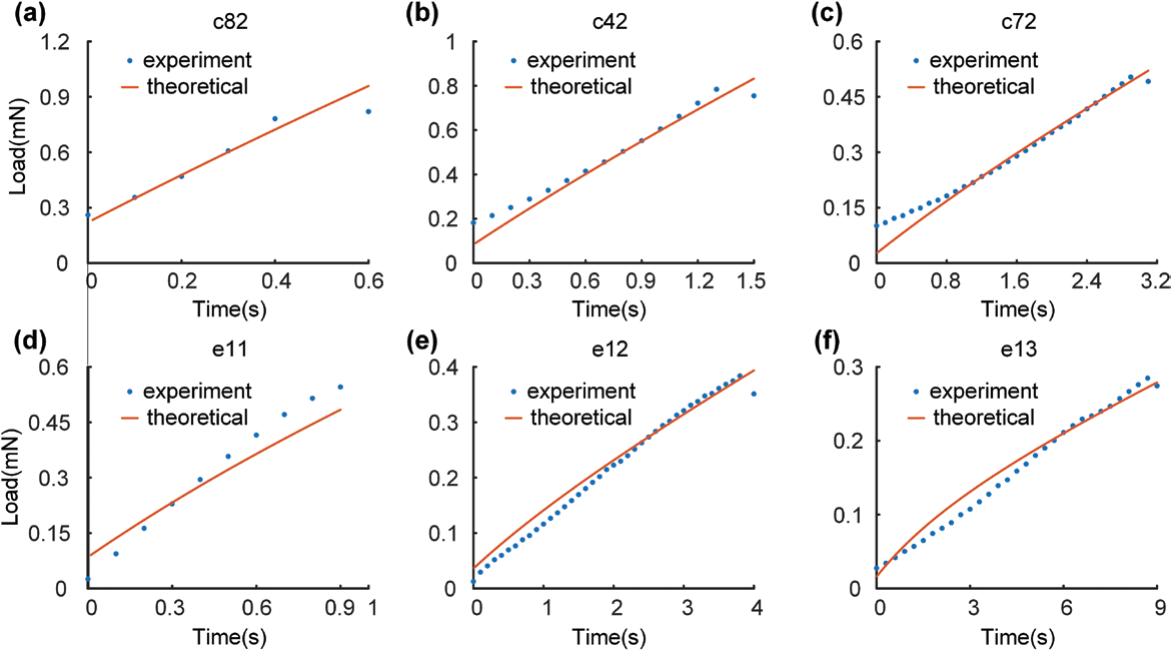
Contrast between experimental load data and theoretical load data calculated with fitting PVM and AEM (in stress relaxation) in the loading process. (a)–(c) In the control group, tests for loading duration of 0.6, 1.5, and 3.2 s. (d)–(f) In the experimental group, tests for loading duration of 1, 4, and 9 s.

The contrast between the theoretical and experimental value of load in the loading process was shown in Fig. 8. The relative error between the theoretical and experimental value was less than 10%. According to Fig. 8a–c in the control group and Fig. 8d and e in the experimental group, the relative error decreased from 10% to 7% with the increase in loading duration. Compared with the relative error of 10% in the research (Van Loocke et al. 2008) of modeling the passive muscle as an entirety the model with PVM and AEM has good fitting performance and is closer to the real structure of muscle.

The main reason for the relative error in the loading process was the Backlash Error of Micro-nano Tensile System. The value of stretching length would decrease a little when the motor stopped $(t={t^{\prime }}_{0})$. Although the decrease was less than 2% of the total stretching length which could be ignored, the duration of the decrease $(\;\delta \;t=t_{0}-{t^{\prime }}_{0})$ was more than 30% of *t*_0_ in the test with a short stretching time. Therefore, the effect of the duration of decrease should be considered. First, experimental data on stress relaxation $\left(t>t_{0}\right)$ involved the unexpected process of stretching length decrease $({t^{\prime }}_{0}<t<t_{0})$ for the Boltzmann superposition principle (Knauss and Zhao 2007). Therefore, this mechanical error caused errors in fitting parameters $(\;\delta \;C_{i}(\delta t),\;\delta \tau_{i}(\delta t),\;\delta A_{i}(\delta t))$ for the deviation during loading. Then the theoretical value of one part of the load in the loading process could be determined by the fitting result causing an error of $Y^{\prime }(t)\;(\;\delta \;Y^{\prime }(\delta C_{i},\delta \tau_{i}))$ according to Equations (2) and (6). Finally, the decrease in stretching length influenced the experimental data during ${t^{\prime }}_{0}<t<t_{0}$, so these data were not used in fitting causing another error of fitting parameters $(\;\delta \;A_{i}(\delta t))$ in AEM. As a result, these errors $(\;\delta \;Y^{\prime }(\delta C_{i},\delta \tau_{i}),\;\delta A_{0}(\delta t))$ caused the error in the theoretical value of load in the loading process according to Equations (7) and (8b).

### Energy Analysis

As the correctness of the model with PVM and AEM verified, the energy storage and release of passive muscle could be expressed through this model by integration of force and deformation (Gau et al. 2019). Since there were 2 parallel modules in the model to present the myosin–titin and cross-bridge–titin structures in passive muscle, the elastic potential energy of passive muscle should be divided into 2 parts related to the 2 modules.

To obtain the analytical solution of energy and analyze the effect of motion parameters on energy storage and release, movement conditions of muscle were given. The passive muscle was stretched to maximum length with a constant velocity of *v*_1_ for duration of *t*_1_ and then recovered with a constant velocity of *v*_2_. The expression of stretching length on the muscle is expressed as $l_{e}(t)=\left\{ \begin{array}{l} v_{1}t\;(0<t\leq t_{1})\\ v_{1}t_{1}-v_{2}t\;(t_{1}<t) \end{array}\right.$.

The first part of the potential energy was determined by the work $E_{e1}$, which was associated with PVM. According to Equation (5), once the stress relaxation coefficient (Y(t)) was confirmed, the load of PVM $\left(F_{PVM}(t)\right)$ could be determined with the given movement of muscle $\left(l_{e}(t)\right)$ (de Vicente 2012). As the initial value of $E_{e1}$ assumed to be zero, substituting the theoretical value of stress relaxation coefficients $\left(Y^{\prime }(t)\right)$ to this integration leads $E_{e1}$ to:


$$\begin{array}{l} E_{e1}(t_{1})=\underset{0}{\overset{t_{1}}{\int }}\;\underset{0}{\overset{t}{\int }}\;Y^{\prime }(t-\tau )v_{1}^{2}d\tau dt,\;\;\;\;\;\;\;\;\;\;\;\;\;\;\;(0<t\leq t_{1}) \end{array}$$ (9)

Besides, the released potential energy in PVM during recovery $\left(t_{1}<t\right)$ was $\Delta E_{e1}(t)=E_{e1}(t)-E_{e1}(t_{1})$, which could be expressed as:


$$\begin{array}{l} \Delta E_{e1}(t)=\underset{t_{1}}{\overset{t}{\int }}\;\left(-\underset{0}{\overset{t_{1}}{\int }}\;Y^{\prime }(t-\tau )v_{1}v_{2}d\tau +\underset{\;t_{1}}{\overset{t}{\int }}\;Y^{\prime }(t-\tau )v_{2}^{2}d\tau \right)dt,\;\;\;(t_{1}<t) \end{array}$$ (10)

To discover the influence of motion parameters $(v_{1},\;v_{1},\;and\;t_{1})$ on energy storage $\left(E_{e1}(t_{1})\right)$ and release $\left(\Delta E_{e1}(t)<0\right)$, we calculated partial derivatives of Equations (9) and (10) with respect to $v_{1},\;v_{1},\;and\;t_{1}$ as follows: $\;\;\;\frac{\partial E_{e1}(t_{1})}{\partial v_{1}}=\underset{0}{\overset{t_{1}}{\int }}\;\underset{0}{\overset{t}{\int }}\;2Y^{\prime }(t-\tau )v_{1}d\tau dt$, $\frac{\partial E_{e1}(t_{1})}{\partial t_{1}}=\underset{0}{\overset{t_{1}}{\int }}\;Y^{\prime }(t_{1}-\tau )v_{1}^{2}d\tau$, $\frac{\partial \Delta E_{e1}(t)}{\partial v_{1}}=\underset{t_{1}}{\overset{t}{\int }}\;\left(-\underset{0}{\overset{t_{1}}{\int }}\;Y^{\prime }(t-\tau )v_{2}d\tau \right)dt$, $\frac{\partial \Delta E_{e1}(t)}{\partial v_{2}}=\underset{t_{1}}{\overset{t}{\int }}\;\left(-\underset{0}{\overset{t_{1}}{\int }}\;Y^{\prime }(t-\tau )v_{1}d\tau +\underset{\;t_{1}}{\overset{t}{\int }}\;2Y^{\prime }(t-\tau )v_{2}d\tau \right)dt$, $\frac{\partial \Delta E_{e1}(t)}{\partial t_{1}}=-\underset{t_{1}}{\overset{t-t_{1}}{\int }}\;Y^{\prime }(\tau )v_{1}v_{2}d\tau -\underset{0}{\overset{t-t_{1}}{\int }}\;Y^{\prime }(\tau )v_{2}^{2}d\tau$.

Due to $Y^{\prime }(t)>0$ with 0 < *t*, the value of $\frac{\partial E_{e1}(t_{1})}{\partial v_{1}}$ and $\frac{\partial E_{e1}(t_{1})}{\partial t_{1}}$ was positive, while the value of $\frac{\partial \Delta E_{e1}(t)}{\partial v_{1}}$ was negative. The signs of $\frac{\partial \Delta E_{e1}(t)}{\partial v_{2}}$ and $\frac{\partial \Delta E_{e1}(t)}{\partial t_{1}}$ were depended on the range of $v_{2}$ and $t_{1}$. Results suggested that the stored elastic potential energy in PVM $\left(E_{e1}(t_{1})>0\right)$ had a positive correlation with stretching velocity $(v_{1})$ and stretching duration $\left(t_{1}\right)$. Meanwhile, the released energy $(\Delta E_{e1}(t)<0)$ had a negative correlation with stretching velocity $(v_{1})$, recovery velocity $(v_{2})$, and stretching duration $\left(t_{1}\right)$ with a low-number range of $v_{2}$ and $t_{1}$.

The second part of the elastic potential energy was $E_{e2}$ depending on AEM. This part of energy depended on the elastic deformation of cross-bridge (Kaya and Higuchi 2010). Since Equation (8b) was constant, the sum force of all cross-bridge was steady in the ramp loading process. Therefore, $E_{e2}$ was a constant value in given loading process $\left(0<t\leq t_{1}\right)$. In modeling, the binding duration of cross-bridge in the cyclical movement was assumed to be Δt, and the time that each cross-bridge had been binding to titin lasted for $\Delta t_{i}\;(1\leq i\leq n)$. According to the cyclical movement of cross-bridge (Jarvis et al. 2021), the deformation of cross-bridge always started at the initial state with no elastic deformation in the load process. Therefore, with Equation (8a), the stored elastic potential energy of AEM in the loading process could be expressed as:


$$\begin{array}{l} E_{e2}=\underset{0}{\overset{1}{\int }}\;\underset{0}{\overset{v_{1}\Delta tx}{\int }}\;\Delta lE_{2}d\Delta ldx,\;\;\;\;\;\;\;\;\;\;\;\;\;\;\;\;\;\;\;\;\;\;\;(0<t\leq t_{1}) \end{array}$$ (11)

In the recovery process $\left(t_{1}<t\right)$, the remaining time for each cross-bridge binding to titin was $\Delta t-\Delta t_{i}$. With equation of $l_{e}(t)$ and the Tension Trigger of Micro-nano Tensile System the released elastic potential energy of the sum of cross-bridge could be expressed as:


$$\begin{array}{l} \Delta E_{e2}=\underset{0}{\overset{1}{\int }}\;\underset{v_{1}\Delta tx}{\overset{v_{1}\Delta tx-v_{2}(\Delta t-\Delta tx)}{\int }}\;\Delta lE_{2}d\Delta ldx,\;\;\;\;(t_{1}+\Delta t\leq t) \end{array}$$ (12)

**Fig. 9.**
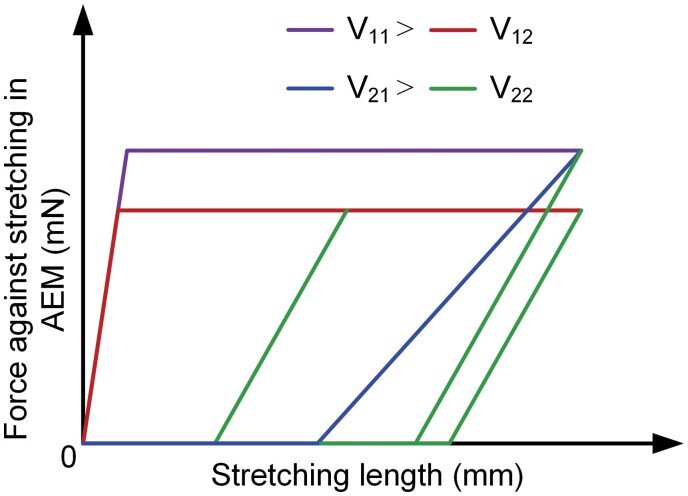
Hysteretic curves of force against stretching in AEM with various stretching and recovery velocities.

After simplifying Equations (11) and (12), energy storage of AEM showed a positive correlation with stretching velocity for $E_{e2}=\frac{1}{6}E_{2}v_{1}^{2}\Delta t^{2}$, while the energy release of AEM had negative correlations with stretching and recovery velocity $\left(v_{2}\leq \frac{v_{1}}{2}\right)$ for $\Delta E_{e2}=\frac{1}{6}E_{2}\Delta t^{2}(v_{2}^{2}-v_{1}v_{2})$. Besides, once the velocities of stretching and recovery were determined, the energy of AEM was determined and did not change with time. It suggested that the sum of the elastic potential energy of cross-bridge achieved a dynamic equilibrium with a constant velocity when stretching. According to Equations (8b), (11), and (12), hysteretic curves of force against stretching in AEM were shown in Fig. 9. In stretching process, the force increased in a fixed track and the force in dynamic equilibrium condition depended on the stretching velocity only. When the muscle was in recovery process, the released elastic energy in cross-bridge increased with the rise of stretching and recovery velocity only.

The above analysis on energy of PVM and AEM showed the same trend for velocity on energy storage and release. It demonstrated that the higher stretching velocity set the more energy stored in muscle during stretching, while the released energy raised with the increase of recovery velocity $\left(v_{2}\leq \frac{v_{1}}{2}\right)$. According to the stress relaxation test, the mechanical work on muscles in the loading process could be calculated with the integral of load on stretching length. Contrasting the work in the same muscle with different motion parameters, the integration of load on stretching length had a positive correlation with stretching velocity and length in Table 1 (*P* < 0.05). Mechanical work on muscles converts into stored energy $(E_{e2}+E_{e1})$ and internal energy (Heat) (Yamada 2017). The increase of mechanical work may cause the increase of elastic energy storage. Therefore, the relationship between energy storage and motion parameters on muscle was the same as the result of the experiment.

**Table 1.** Contrast of mechanical work on muscles in loading process with different motion parameters

Test number	Stretching velocity (mm/s)	Stretching length (mm)	Work (µJ)
c61	0.2	0.1959	0.04371
c62	0.1	0.197	0.03352
c81	0.2	0.1591	0.1735
c82	0.2	0.0794	0.03884
e22	0.1	0.1683	0.03687
e23	0.05	0.1627	0.0357
e51	0.05	0.1926	0.02614
e52	0.05	0.1276	0.0123

This relationship between energy storage/release and motion parameters on muscle suggests that the stretching velocity and length should be larger to get more energy stored in PVM, while just stretching velocity needs to be larger to store more energy in AEM. Besides, the released energy raised with the increase of recovery velocity at a low-number range. This relationship was confirmed with the experimental data in the loading process and other species like frogs (Linari et al. 2003) and rabbits (Fukutani et al. 2017).

### The Application of the Model on Changes of Muscle Structures

According to this model, the load drop in rapid stress relaxation stages corresponds with the release of elastic force from cross-bridge in AEM and viscoelastic force from titin–myosin in PVM, while the load drop in slow stage corresponds with the latter only. Besides, according to Expressions (2) and (4) of force from AEM and PVM in stress relaxation in the Statistics and Data Processing section, the 2 forces are linear correlation with the stretching velocity. As Fig. 3a and b showed, the ratio of load drops had a positive correlation with the stretching velocity. However, with the increase of stretching velocity, the slopes of load-drop ratio decreased. It suggests that with the increase of stretching velocity, the release of elastic force in AEM may gradually reach the maximum for the extreme elastic deformation of cross-bridge. The elastic deformation of cross-bridge may be based on the connection between cross-bridge and titin. Since there is an ultimate strength on this connection (Kaya and Higuchi 2010). According to the expression of the elastic force of cross-bridge in AEM in loading process in the Verification of the Model section, after the stretching velocity reaches a certain value, the elastic force of cross-bridge will achieve the ultimate strength of the connection and the connection will break to release the force. Then no matter what the stretching velocity increase, the elastic force of cross-bridge will keep the maximum at the ultimate strength of this connection. It indicates that the ultimate strength on the connection between cross-bridge and actin limits the maximum elastic deformation of cross-bridge in AEM. Unfortunately, there is no method to gain the exact number of the connecting cross-bridge in each sample unless using Laser Trap in single actin (Kaya et al. 2017, Pertici et al. 2018), so the ultimate strength on this connection is unable to be calculated.

**Fig. 10.**
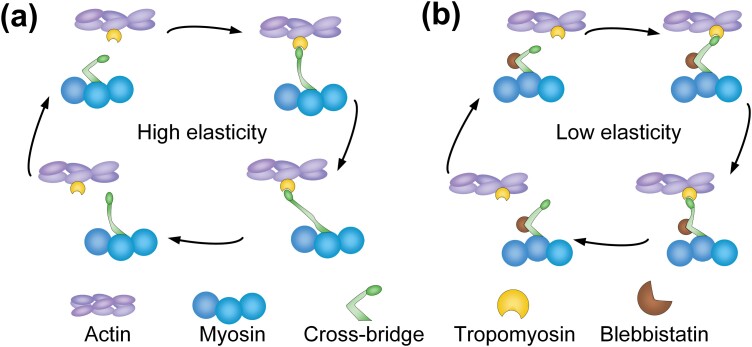
The effect of blebbistatin on the elastic of cross-bridge. (a) High elasticity of cross-bridge without blebbistatin in stretching. (b) Low elasticity of cross-bridge combined with blebbistatin in stretching.

Fitting results and verification in the loading process proved the correctness of the model. This suggests that considering the muscle as a parallel structure composed of a tandem myosin–titin viscoelastic base and numerous separate cross-bridge–actin cyclic connections is more consistent with the real structure of muscle than considering it as an entirety (Meyer et al. 2011, De Vita et al. 2017, Wheatley 2020). Hence, the difference of $nE_{2}$ between control and experimental groups in AEM in the Model Fitting section may reveal the effect of blebbistatin on the elastic of cross-bridge. As the expression of $nE_{2}$, the decrease of $nE_{2}$ with adding blebbistatin suggests that the stiffness (*E*_2_) or amount (*n*) of connecting cross-bridge could decline. The change of *E*_2_ indicates that after adding blebbistatin, the stiffness of cross-bridge decreases for the combination of cross-bridge and blebbistatin, which is in accord with the effect of blebbistatin (Lemaire et al. 2019). Besides, the change of *n* suggests that blebbistatin influences the connection of cross-bridge with actin. The connection point on cross-bridge for blebbistatin is located between connection points for actin and ATP (Kovács et al. 2004). Since the combination of blebbistatin, the hydrolysis and separation of ATP is restricted (Kovács et al. 2004), causing the disappearance of the stroke of cross-bridge in active muscle and the change of the cycle movement between cross-bridge and actin. Fig. 10 showed the influence of adding blebbistatin to muscle in the relaxation test. With the combination between cross-bridge and blebbistatin, the capacity of deformation on cross-bridge was limited in the whole process of tests, while the myosin–titin series was not affected.

This model could also explain the change of relaxation curve for the change of muscle structure in other relaxation researches. In Linke and Leake (2004), after the actin peeled from muscles, the load drop at the beginning of relaxation curve changes from large to small. According to the model in this study, the reason seems that the connection cycle between cross-bridge and actin disappears for the peeled actin. Without the connection cycle, AEM in this model should be ignored causing the disappearance of the load drop of AEM in the rapid stress relaxation stage. Therefore, the load drop before actin peeled is the sum of load drops in AEM and PVM, which is larger than the only load drop of PVM after the actin peeled from muscles. In Caporizzo et al. (2020), the relaxation curve reduces in overall load with colchicine treated on muscles to dissolute the microtubule. However, there is little change in the load drop in stress relaxation, especially in the rapid stress relaxation stage. Besides, the structural feature of microtubules in muscles might be similar to the series of myosin and titin for microtubule spreading throughout the sarcomere (Bruno et al. 2011) as the same as the distribution of myosin–titin series. According to the model, the microtubule should be a part of PVM, so the dissolution of the microtubule decreases the viscoelastic modulus of PVM causing a decrease in overall load. However, the dissolution of the microtubule does not influence AEM meaning the constant load drop of AEM in the rapid stress relaxation stage. Therefore, the load drops show no difference between control and colchicine groups in the rapid stress relaxation stage with a high stretching velocity. Besides, according to the stiffness changes in titin–myosin and cross-bridge–actin structures from passive to active muscle (Zhang et al. 2021), this model may also be used for studying the biomechanisms of active muscle by changing the stiffness-related coefficient.

Stress relaxation tests performed on fresh abdominal muscles of honey bees showed that there was a rapid stage depending on stretching velocity and stretching length and a slow stage depending on stretching length in the load drop of stress relaxation. The 2 stages reflect the structure of the myosin–titin base and cross-bridge–actin cyclic connections in muscles. According to the analysis of results, the model with 2 parallel modules (PVM and AEM) was improved to fit the data in stress relaxation tests. This model considered the mechanical properties and behavioral characteristics of different structural proteins in honey bee muscles to distinguish the different effects on muscle energy storage. The model fitted well with the experimental data in the stress relaxation stage and loading process. Changes in the fitting parameter in AEM with different concentrations of blebbistatin proved the stiffness or connecting number of cross-bridge would decrease with a combination of blebbistatin. Besides, expressions of energy storage and release on passive muscle were deduced, and consistent with the experimental data. The result suggests that with a larger velocity of cycle movement on the honey bee abdomen, the more energy would be stored and released for the elastic deformation of cross-bridge.

## Supplementary Material

iead026_suppl_Supplementary_Table
