# Supplementary material for: Mechanism of elastic energy storage of honey bee abdominal muscles under stress relaxation
Source: J Insect Sci. 2023 May 8;23(3):2. doi: 10.1093/jisesa/iead026 (PMC10205004; doi:10.1093/jisesa/iead026)
Supplement: iead026_suppl_Supplementary_Table [file iead026_suppl_supplementary_table.docx]

**Appendixes A**

**Table A1**

Motion parameters of each stress relaxation test.

(a) Control group

| Test number | stretching velocity (mm s^-1^) | stretching length (mm) |
| --- | --- | --- |
| c11 | 0.03 | 0.0848 |
| c12 | 0.02 | 0.0417 |
| c13 | 0.01 | 0.0386 |
| c21 | 0.2 | 0.0588 |
| c22 | 0.1 | 0.0983 |
| c23 | 0.02 | 0.0455 |
| c31 | 0.2 | 0.0789 |
| c32 | 0.05 | 0.0932 |
| c33 | 0.02 | 0.4365 |
| c41 | 0.2 | 0.0188 |
| c42 | 0.1 | 0.1283 |
| c43 | 0.05 | 0.0081 |
| c51 | 0.05 | 0.0279 |
| c61 | 0.2 | 0.1959 |
| c62 | 0.1 | 0.197 |
| c71 | 0.1 | 0.148 |
| c72 | 0.05 | 0.1426 |
| c81 | 0.2 | 0.1591 |
| c82 | 0.2 | 0.0794 |
| c91 | 0.05 | 0.1781 |
| c92 | 0.05 | 0.1134 |

(b) Experimental group

| Test number | stretching velocity (mm s^-1^) | stretching length (mm) |
| --- | --- | --- |
| e11 | 0.2 | 0.1796 |
| e12 | 0.05 | 0.1882 |
| e13 | 0.02 | 0.174 |
| e21 | 0.2 | 0.1789 |
| e22 | 0.1 | 0.1683 |
| e23 | 0.05 | 0.1627 |
| e31 | 0.2 | 0.0585 |
| e32 | 0.1 | 0.2074 |
| e33 | 0.05 | 0.2028 |
| e41 | 0.2 | 0.1792 |
| e42 | 0.2 | 0.1398 |
| e51 | 0.05 | 0.1926 |
| e52 | 0.05 | 0.1276 |
